# Enhancing Received Signal Strength-Based Localization through Coverage Hole Detection and Recovery †

**DOI:** 10.3390/s18072075

**Published:** 2018-06-28

**Authors:** Shuangjiao Zhai, Zhanyong Tang, Dajin Wang, Qingpei Li, Zhanglei Li, Xiaojiang Chen, Dingyi Fang, Feng Chen, Zheng Wang

**Affiliations:** 1 School of Information Science and Technology, Northwest University, Xi’an 710127, China; sjzhai@stumail.nwu.edu.cn (S.Z.); lqp@stumail.nwu.edu.cn (Q.L.); xueyzhai@gmail.com (Z.L.); xjchen@nwu.edu.cn (X.C.); dyf@nwu.edu.cn (D.F.); xdcf@nwu.edu.cn (F.C.); 2 School of Computer Science, Montclair State University, Montclair, NJ 07043, USA; wangd@montclair.edu; 3 School of Computer Science & Technology, Xi’an University of Posts & Telecommunications, Xi’an 710121, China; z.wang@lancaster.ac.uk; 4 School of Computing and Communications, Lancaster University, Lancaster LA1 4WA, UK

**Keywords:** wireless sensor networks, RSSI-based localization, coverage holes, Voronoi tessellation, Delaunay triangulation

## Abstract

In wireless sensor networks (WSNs), Radio Signal Strength Indicator (RSSI)-based localization techniques have been widely used in various applications, such as intrusion detection, battlefield surveillance, and animal monitoring. One fundamental performance measure in those applications is the sensing coverage of WSNs. Insufficient coverage will significantly reduce the effectiveness of the applications. However, most existing studies on coverage assume that the sensing range of a sensor node is a disk, and the disk coverage model is too simplistic for many localization techniques. Moreover, there are some localization techniques of WSNs whose coverage model is non-disk, such as RSSI-based localization techniques. In this paper, we focus on detecting and recovering coverage holes of WSNs to enhance RSSI-based localization techniques whose coverage model is an ellipse. We propose an algorithm inspired by Voronoi tessellation and Delaunay triangulation to detect and recover coverage holes. Simulation results show that our algorithm can recover all holes and can reach any set coverage rate, up to 100% coverage.

## 1. Introduction

In wireless sensor networks (WSNs), a thorough coverage of the target regions is of vital importance to the performance of its applications. For example, in activity tracking, knowing which areas are not covered by sensors would allow one to deploy additional sensors or paying attentions to the coverage holes [1]. Coverage holes could have many causes, including sensor faults, a sub-optimal sensor deployment, and unanticipated errors after deployment.

**Figure 1:**
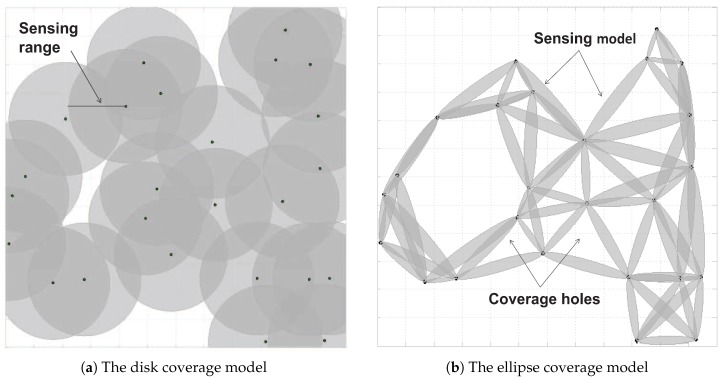
WSN coverage models. (**a**) The centre of a disk is the sensor node coordinates. Its radius is the sensing range of the sensor node. (**b**) The ellipse coverage model of WSNs. The ellipse coverage model is given in the first Fresnel zone of two communicating sensor nodes.

A large number of studies have been devoted to detecting and recovering coverage holes [2,3,4,5,6,7,8,9,10,11,12,13,14,15]. However, most existing approaches [2,3,4,5,6,7,8,9,16,17,18,19,20,21,22] are based on an assumption that the sensing range of a sensor node is like a disk (as shown in Figure 1a) with the centre of the disk as a sensor. Unfortunately, this assumption does not hold in many localization scenarios. For example, this assumption does not hold for a Radio Signal Strength Indicator (RSSI)-based localization application [23,24,25,26,27,28], whose task is to locate objects according to the disturbance of the objects to several communication links. This is because the coverage model of RSSI-based localization techniques is an ellipse [25] instead of a disk. This is illustrated in Figure 1b, in which the single gray range is the coverage model determined by a pair of sensor nodes, and the Euclidean distance of two sensor nodes is less than the transmission range of sensors. Due to the structural differences, existing coverage hole recovering methods based on the disk model is not applicable to RSSI-based location. This is because there still exist coverage holes for the ellipse coverage model, even though it is full coverage for the disk coverage model. As shown in Figure 1, a total of 25 sensor nodes are randomly deployed in a 300×300 m region. Figure 1a,b show the total coverage regions based on the disc coverage model and the ellipse coverage model, respectively, and these sensor nodes have the exact same sensing range. Obviously, Figure 1b has more coverage holes than Figure 1a. More details will be dealt with in Section 6.2. Furthermore, compared to WSNs with the disk coverage model, WSNs with the ellipse coverage model are even more vulnerable to coverage holes due to the fact that the coverage model is determined by two sensors instead of one, and failure of any one of the two sensors could lead to coverage holes.

In this paper, we propose an algorithm to detect and recover coverage holes of WSNs based on RSSI-based localization techniques whose coverage model is an ellipse [25]. The algorithm is based on Voronoi tessellation and Delaunay triangulation. More precisely speaking, we recover coverage holes by adding additional sensors, and the locations of these additional sensors are determined by Voronoi tessellation and Delaunay triangulation. Thus, in order to recover coverage holes, how to deploy additional sensors as little as possible has been the biggest challenge. We call the problem the Optimum Increase Coverage Problem (OICP), i.e., deploying only a few additional sensors to increase the coverage rate as much as possible. In this paper, we first investigate the ellipse coverage model of RSSI-based localization techniques from the perspective of theory and experiment, respectively. Next, stimulated by Voronoi tessellation and Delaunay triangulation, an approximation algorithm is proposed to detect and recover coverage holes. This algorithm will be shown in detail in Section 5.

The main contributions can be summarized as follows:

- To the best of our knowledge, this is the first paper to detect and recover coverage holes of WSNs based on RSSI-based localization techniques, and we will enhance RSSI-based localization through coverage hole recovery.
- We systematically investigate the coverage model of RSSI-based localization techniques, and ellipse coverage model is derived from theoretical analysis and experimental verification.
- An approximation algorithm is proposed to recover coverage holes in WSNs with ellipse coverage model. Simulation results show that our algorithm can recover all holes and can reach any set coverage rate, up to 100% coverage.

The rest of this paper is structured as follows. Related work is described in Section 2. We provide background in Section 3. Section 4 justifies the ellipse coverage model of RSSI-based localization techniques. An approximation algorithm to detect and recover coverage holes are detailed in Section 5. We present experimental results and performance evaluation in Section 6. Lastly, we conclude this paper in Section 7.

## 2. Related Work

Our work builds upon the following techniques, while qualitatively differing from each.

### 2.1. RSSI-Based Localization Techniques

RSSI-based localization techniques are widely used for intrusion detection, battlefield surveillance, animal monitoring, etc. [23,24,25,26,27,28,29,30,31,32,33,34,35]. One of the important applications of RSSI-based localization techniques is monitoring and tracking wildlife [36,37]. Liu et al. [36] designed a monitoring system to locate and track Rhinopithecus roxellana. Dyo et al. [37] designed a RFID–WSN hybrid system to investigate the social behaviour of European badgers in a forest. These examples show that RSSI could be a viable means for wildlife tracking. RSSI-based localization techniques have numerous advantages [38], as they require no additional hardware, and the energy consumption is relatively low. While promoting, the performance of RSSI-based methods will be greatly degraded when the effective wireless signals do not cover the entire target region. Our work offers a new way to enhance RSSI-based localization by detecting coverage holes.

### 2.2. Coverage Hole Detection and Recovery

There is an extensive body of work on coverage hole detection and recovery for WSNs [2,3,4,5,6,7,8,9,16,17,18,19,20,21,22]. Bi et al. [16] proposes a distributed system to detect coverage holes based on the communication topology graph. However, the proposed approach only works for a relatively small WSN with small coverage holes. Ghrist et al. [17] and Silva et al. [18] detect coverage holes using an analytical model. While effective, the proposed model has a high complexity, preventing it from being applied to large WSNs due to the overhead for collecting the require information. In this paper, we propose a geometrical approach to detect coverage holes, which has a lower complexity and can be effective for less dense nodes.

There are also methods based on Delaunay triangulation. Wu et al. [39] propose a centralized sensor deployment method, DT-Score, to deploy sensors in a given sensing area with obstacles. In the first phase, sensors are deployed near the boundary of the target area and obstacles. In the second phase, a method based on the Delaunay triangulation is put forward to recover coverage holes. In [40], a method based on the theory of trees and graphs is proposed recover coverage holes. However, all of these works are inapplicable RSSI-based localization, whose coverage model is an ellipse determined by two communicating sensors. The aforementioned methods are based on an assumption that the coverage model of WSNs is a disk. This assumption is shown to be invalid for RSSI-based location [6]. As a departure from prior work, we propose a novel coverage hole detection and recovery method for the ellipse coverage model.

## 3. Background

**Figure 2:**
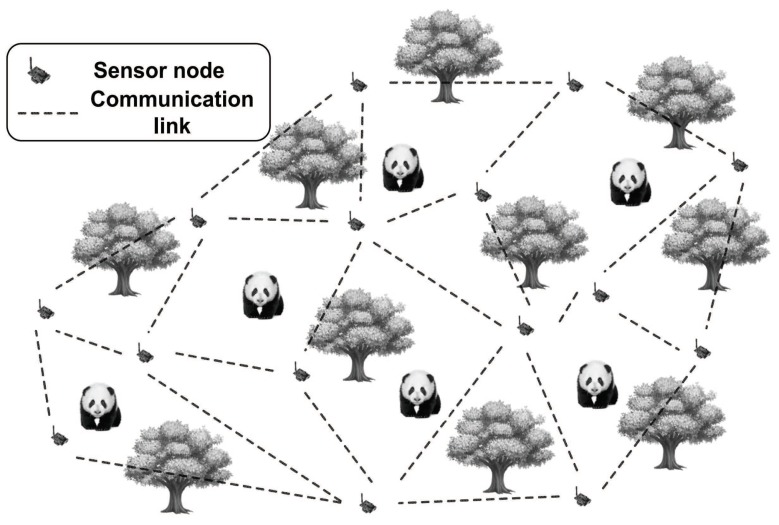
Illustration of RSSI-based localization techniques. A number of sensors are deployed in advance in the activity area of giant pandas in a wildlife park, and giant pandas are the objects of interest. These sensors continuously transmit packets and measure the RSSI value of the corresponding communication links. When pandas are present, additional communication links will be introduced between sensors (this is also referred as the multi-path effects). As a result, the RSSI value will be considerably changed. Combined with the related algorithms, the locations of giant pandas can be estimated.

Our work exploits wireless sensors to detect activities by monitoring how the RSSI signal is affected by a moving object. As an example, Figure 2 depicts how RSSI-based object localization can be deployed to track and monitor giant pandas. In this scenario, the present of a giant pandas will change the RSSI signal pattern between sensors, which is then matched against training samples to identify if a panda was appearing in the target area. Our goal is to identify the coverage holes of the deployment site, i.e., where the wireless signal is not covered.

In this work, we assume that there is only one target object in the deployment area at any given moment. Multi-object localization using RSSI is an open problem, which is beyond the scope of this work. To eliminate the impact of subtle environmental changes such as moving trees, we use machine learning to filter out the disturbance of the environment. H. Ahmadi et al. [35] proposes a novel target tracking algorithm, which is based on regression tree and filtering methods. The evaluation results show that the proposed method is robust to noisy environment and environmental change.

## 4. The Ellipse Coverage Model

In this section, we will analyze the coverage model of RSSI-based localization techniques from the perspective of theory and experiment, respectively. The detailed derivation of the ellipse coverage model will be given. Table 1 shows the main notations used in this paper.

### 4.1. Theoretical Model

Sensors in WSNs are used as both receivers and transmitters. When sensors communicate with each other, there are wireless signals between them, and these wireless signals are essentially electromagnetic waves. According to the theory of electromagnetic wave propagation and RSSI-based localization techniques, when the electromagnetic waves encounter an object, they will be diffracted, scattered, absorbed, or reflected. Thus, the RSSI value of the corresponding communication links will be changed, and the objects will be tracked and monitored based on these changes.

Therefore, we next analyze the coverage model from the perspective of electromagnetic waves. It is a known fact that sensors transmit electromagnetic waves through the way of line-of-sight (LOS) and non-line-of-sight (NLOS). In particular, for the way of LOS, sensors transmit electromagnetic waves mainly along a straight line like light transmission. Whereas for the way of NLOS, sensors transmit electromagnetic waves by diffracting and scattering. In this paper, we only pay attention to the scene of NLOS.

Firstly, influences of scattering will not to be analyzed. This is because in the ionosphere, there are some non-uniform particles (e.g., electrons) whose volume is much smaller than the wavelength of electromagnetic waves, and the electromagnetic waves will be scattered or reflected by it. Thus, part of the electromagnetic waves cannot reach receiving sensors along a straight line. At the same time, rough surfaces such as trees will also scatter electromagnetic waves to some other positions, so signals received from sending sensors will gain. However, the scattering signals are very weak, and it is almost undetectable unless special equipment is used. Thus, under the background of WSNs, whose sensors are only just some common sensors, influences of scattering are especially low, so it can be ignored.

**Figure 3:**
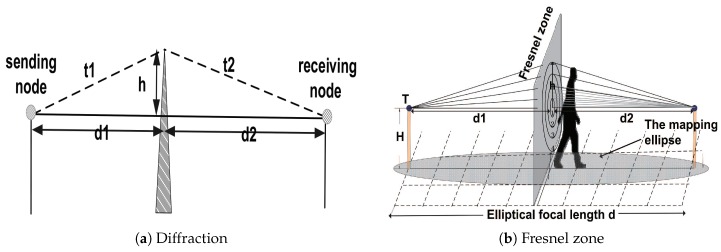
Diffraction and Fresnel zone. (**a**) The diffraction of electromagnetic waves. There is a knife-edge obstacle between sending sensor and receiving sensor, its relative height is *h*, and distances from two sensor nodes are $d_{1}$ and $d_{2}$, respectively. The propagation distance of electromagnetic wave “bypasses” the knife-edge obstacle and is $t=t_{1}+t_{2}$. (**b**) The Fresnel zone. The Fresnel zone is a series of concentric circles consisting of different ellipsoids. For any cross section *s*, from inside to outside of the concentric circle, it can be defined as the 1st, 2nd, 3rd, …, *n*th Fresnel zone in turn.

Instead, diffraction needs to be taken into consideration. Diffraction refers to a situation where there are obstacles between sending sensors and receiving sensors, and where the volume of the obstacles is smaller than the wavelength of electromagnetic waves or has a sharp edge that is comparable with the wavelength. The electromagnetic waves can “bypass” the obstacles by means of diffraction. The frequency of antenna is usually 2.4 GHz, so the wavelength can be calculated by formula v=λ·f, in which $v=3\times 10^{8}$ m/s . As a result, λ = 0.125 m, and this is a large range. Therefore, it is indispensable to take diffraction into consideration for object localization. An example of diffraction is shown in Figure 3a. Assuming that there is a knife-edge obstacle between sending sensors and receiving sensors, its relative height is *h*, and distances from two sensor nodes are $d_{1}$ and $d_{2}$, respectively. The propagation distance of electromagnetic waves “bypasses”the knife-edge obstacle and is $t=t_{1}+t_{2}$. It is obvious that the propagation distance of the diffracted wave is greater than the direct wave, which can produce a phase difference between these two waves, can form the wave interference or overlay and eventually lead to changes in the RSSI value.

According to Chen Liu et al. [36], Fresnel zones can be introduced to model the phenomenon of diffraction. In geometry, Fresnel zone is a series of concentric circles consists of different ellipsoids. As shown in Figure 3b, for any cross section *s*, from inside to outside of the concentric circle, it can be defined as the 1st, 2nd, 3rd, …, *n*th Fresnel zone in turn. Thus, radius of the *n*th Fresnel zone can be obtained by Equation (1), where $d_{1}$ and $d_{2}$ are the distance from the cross section of the Fresnel zone to the sending node and the receiving node respectively, and λ is the wavelength of the electromagnetic wave. In terms of the diffraction theory [41], propagation of the electromagnetic wave is mainly completed in the first Fresnel zone. Therefore, as long as the first Fresnel zone is not blocked, it can be thought that RSSI value between two sensor nodes is not affected.

(1) $$r_{n}=\sqrt{\frac{n\lambda \cdot d_{1}\cdot d_{2}}{\left(d_{1}+d_{2}\right)}}.$$

**Figure 4:**
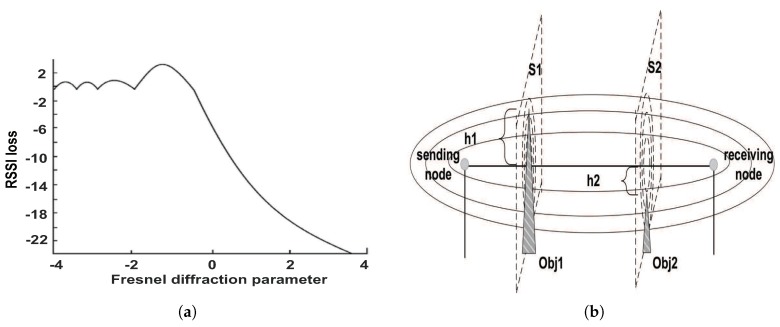
Fresnel diffraction. (**a**) Relationships between diffraction parameter *v* and RSSI loss. When v<−1, RSSI loss is almost zero. This means that the obstacle hardly has any effect on the RSSI value when it does not affect the first Fresnel zone. (**b**) The impact of different obstacles. $Obj_{1}$ and $Obj_{2}$ are two obstacles, whose relative height to the sensor nodes is $h_{1}$ and $h_{2}$, respectively, and $h_{2}$ is a negative number.

Next, we further verify this theory. According to [42], the diffraction effect of the electromagnetic wave is given by Equation (2), where F(v) is a function of *v*. In addition, *v* is the Fresnel diffraction parameter that can be calculated by Equation (3), where $d_{1}$ and $d_{2}$ are the distance from the obstacle to the sending node and the receiving node, respectively, λ is the wavelength of the electromagnetic wave, and *h* is the relative height of the obstacle to the sending node and the receiving node. Relationships between the diffraction parameter and the RSSI loss (also referred to as changes in the RSSI value) are given by Rappaport [41]. We intuitively illustrate the relationships through a figure shown in Figure 4a. As shown in Figure 4a, the diffraction effect, i.e., RSSI loss, is the largest when the Fresnel diffraction parameter v=0. Furthermore, according to Equation (3), when v=0, h=0. This also means that the obstacle has the largest diffraction effect when its sharp edge in the middle of the first Fresnel zone. Similarly, Figure 4a shows that, when v<−1, i.e., *h* is less than a certain value according to Equation (3), RSSI loss is almost zero. This means that the obstacle hardly has any effect on the RSSI value when it does not affect the first Fresnel zone. An example is used to explain it, which is shown in Figure 4b. Two obstacles are recorded as $Obj_{1}$ and $Obj_{2}$, respectively, whose relative height to the sensor nodes is $h_{1}$ and $h_{2}$, respectively, and $h_{2}$ is a negative number. According to Figure 4a and Equation (3), $Obj_{1}$ has an effect on the RSSI value of the communication link of two sensors, while $Obj_{2}$ does not. Therefore, to sum up, in addition to the first Fresnel zone on one communication link, the obstacle in other regions has no obvious effect on the RSSI value. Thus, when mapping the first Fresnel zone on a two-dimensional plane, it is an ellipse.

(2) $$G_{d}=20lg\left|F(v)\right|$$

(3) $$v=h\sqrt{\frac{2\cdot (d_{1}+d_{2})}{\lambda d_{1}d_{2}}}.$$

### 4.2. Mathematic Model

Suppose that all sensor nodes have the same transmission range $d_{max}$, and these sensor nodes are randomly deployed in the target regions, which will not be moved if they have been deployed. Every sensor node is aware of its location either via GPS or with the help of other localization methods. Note that the target regions are a 2-dimensional plane denoted as $R^{2}$, we construct a Cartesian coordinate system based on its lower left corner as the origin. Let $s_{i}$ represent the *i*-th sensor, and $s_{i}$($x_{i}$, $y_{i}$) its coordinate. Thus, *S* = { $s_{1}$, $s_{2}$, $s_{3}$, …, $s_{n}$ } is the set of sensors in the target regions, and *n* is the number of sensors in $R^{2}$.

For any two sensor nodes in the target regions, denoted as $s_{i}$ and $s_{j}$, whose coordinates are $s_{i}$($x_{i}$, $y_{i}$) and $s_{j}$($x_{j}$, $y_{j}$), respectively, only when the Euclidean distance $d_{ij}$ between them is equal to or less than the transmission range $d_{max}$, i.e., $d_{ij}$⩽$d_{max}$, can they communicate with each other and form an ellipse coverage model. According to the first Fresnel zone, the focal length of the ellipse is $d_{ij}$, its long axis can be considered $d_{ij}$ when $d_{ij}$ is especially large. The short axis of the ellipse can then be denoted as $\sqrt{\lambda d_{ij}}$ calculated by Equation (1). Thus, the ellipse can be calculated by Equation (4), where $d_{ij}$ is the Euclidean distance between two communicating sensors, λ is the wavelength of the electromagnetic wave, ($x_{i}$, $y_{i}$) and ($x_{j}$, $y_{j}$) are the coordinates of two communicating sensors, and θ is the counterclockwise rotation angle. sinθ and cosθ can be calculated by Equation (5). Equation (4) is deduced from Equation (6), which is an arbitrary elliptic parameter equation in the 2-dimensional plane.

(4) $$\left\{\begin{array}{c} x=d_{ij}\times \cos t\times \cos\theta -\sqrt{\lambda d_{ij}}\times \sin t\times \sin\theta +\frac{x_{i}+x_{j}}{2}\\ y=d_{ij}\times \cos t\times \sin\theta +\sqrt{\lambda d_{ij}}\times \sin t\times \cos\theta +\frac{y_{i}+y_{j}}{2} \end{array}\right.$$

(5) $$\sin\theta =\left|\frac{y_{j}-y_{i}}{d_{ij}}\right|,\cos\theta =\left\{\begin{array}{c} \frac{x_{i}-x_{j}}{d_{ij}},y_{i}>y_{j}\\ \frac{x_{j}-x_{i}}{d_{ij}},y_{i}<y_{j} \end{array}\right.$$

(6) $$\left\{\begin{array}{c} x=\;a\times \cos t\times \cos\theta -b\times \sin t\times \sin\theta +X\\ y=\;a\times \cos t\times \sin\theta +b\times \sin t\times \cos\theta +Y \end{array}\right..$$

### 4.3. Experimental Verification

**Figure 5:**
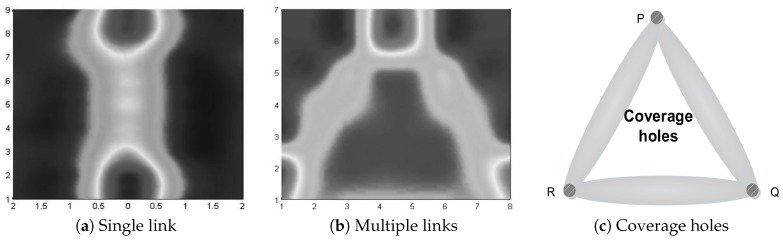
Experimental results in a single link (**a**) and multi-link (**b**) scenarios, together with a coverage hole illustration (**c**).

We conducted experiments in an outdoor environment and analyzed coverage models for scenarios of a single link and multiple links, respectively. Figure 5 summarizes the results (detailed discussions are given in Section 6.1). Figure 5a shows the result of a single link, and the different gray scales stand for different changes of RSSI values. The result shows that the changes in RSSI values are negligible when the person stands in a cell more than a certain distance away from the connection line of two sensors. Furthermore, it also proves that the ellipse can be used as a coverage model of RSSI-based localization techniques. Figure 5b shows the result of multiple links. Similarly, the different gray scales stand for different changes in RSSI values. Thus, the regions in the centre of the three sensors are the coverage holes. Figure 5c shows the coverage holes in theory. Obviously, coverage holes in theory and experiment are similar.

## 5. Detecting and Recovering Coverage Holes

**Figure 6:**
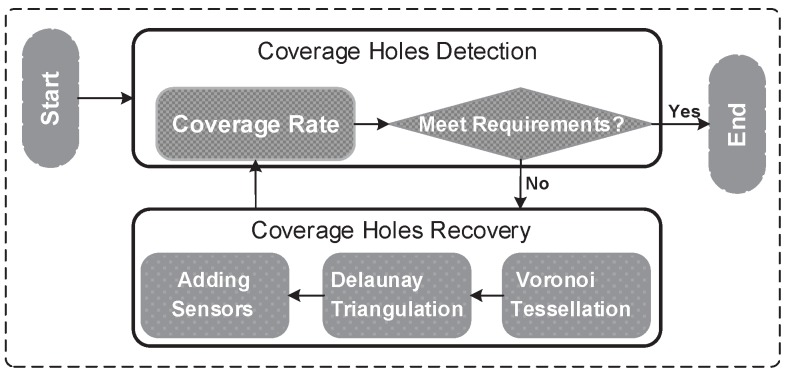
Implementation of the algorithm.

In this section, we propose an algorithm to detect and recover coverage holes. Firstly, coverage holes are detected by calculating the coverage rate. We believe that there are coverage holes as long as the coverage rate does not meet the requirement of the corresponding application. We then recover coverage holes by deploying additional sensors. Deployment locations of the additional sensors are determined by Voronoi tessellation and Delaunay triangulation. Figure 6 shows the implementation of the algorithm.

### 5.1. Detecting Coverage Holes

In order to calculate the coverage rate, the total area of the ellipses in the target regions needs to be calculated in the first instance. According to the description of the ellipse coverage model in Section 4.2, let $F=\left\{f_{ij}|i,j=1,2,\ldots ,n,i\neq j\right\}$ denote the set of ellipses, where $f_{ij}$ is an ellipse determined by $s_{i}$ and $s_{j}$. Only if the Euclidean distance between $s_{i}$ and $s_{j}$ is equal to or smaller than $d_{max}$ can the ellipse $f_{ij}$ be formed. According to Equation (4), the ellipse can be modeled. However, the total area of ellipses will be increased when we add up all $f_{ij}$ in *F*. This is because there are overlaps between ellipses formed by different sensors. Therefore, we calculate whether a point is covered, which can be calculated according to Equation (7), where $x_{p}$ and $y_{p}$ are the horizontal coordinate and the vertical coordinate of the point, and *X* and *Y* denote an ellipse in the target regions calculated by Equation (4). The point is covered when it meets Equation (7) at least once, i.e., the point is within at least an ellipse. Let $p_{covi}$ denote that a point can be covered by *i* ellipses. When i>0, let $p_{covi}=p_{cov1}$, that is, $p_{cov0}$ means that the point cannot be covered by any ellipse. Therefore, the total area of the ellipses is to add all points that are $p_{cov1}$.

However, it is not necessary to calculate the coverage of a single point in the target regions for a practical application. This is because the object will occupy some regions. Thus, we partition the target regions into several small enough square elements, i.e., $R^{2}=\left\{a_{1},a_{2},a_{3},\ldots ,a_{m}\right\}$, where $a_{i}$ is a square element that consists of $y_{1}=x_{i},y_{2}=x_{i+\Delta h},x_{1}=y_{i},x_{2}=y_{i+\Delta h}$,and it is covered when its centre is within an ellipse, i.e., $m\left(\frac{x_{i}+x_{i+\Delta h}}{2},\frac{y_{i}+y_{i+\Delta h}}{2}\right)$ is $p_{cov1}$. Let $p_{a_{i}}$ denote the coverage of the square element $a_{i}$. Thus, $ℜ=\left\{p_{a_{1}},p_{a_{2}},p_{a_{3}},\ldots ,p_{a_{t}}\right\}$ denotes the set of covered square elements. Therefore, the coverage rate of the target regions can be obtained by calculating the ratio of the number of elements in the collection *ℜ* with the number of square elements in the target regions. The size of a square element and the threshold of the coverage rate can be determined by the specific application and the volume of the target. When the coverage rate is lower than this threshold, it is considered that there are coverage holes in the target regions. The pseudocode of detecting coverage holes is shown in Algorithm 1.

(7) $$\left\{\begin{array}{c} x=\left(x_{p}-X\right)\times \cos\theta +\left(y_{p}-Y\right)\times \sin\theta \\ y=\left(X-x_{p}\right)\times \sin\theta +\left(y_{p}-Y\right)\times \cos\theta \\ \frac{x^{2}}{{\left(\frac{d_{ij}}{2}\right)}^{2}}+\frac{y^{2}}{{\left(\frac{\sqrt{\lambda d_{ij}}}{2}\right)}^{2}}⩽1 \end{array}\right..$$

**Algorithm 1:** Procedure of Detecting Coverage Holes**Input**:  The set of ellipses: $F=\left\{f_{ij}|i,j=1,2,\ldots ,n,i\neq j\right\}$; The set of square elements partitioned in the target regions: $R^{2}=\left\{a_{1},a_{2},a_{3},\ldots ,a_{m}\right\}$; The total number of covered square elements *P* is initiated as 0;
**Output**: coverage_rate;
**1** **for** *each square element $a_{i}\in R^{2}$* **do****2**   **for** *each ellipse $f_{ij}\in F$* **do****3**     **if** *the centre of $a_{i}$ inside the ellipse $f_{ij}$* **then****4**       P=P+1;**5**       return;**6**     **end****7**   **end****8** **end****9** coverage_rate=P/m;

### 5.2. Recovering Coverage Holes

When the coverage rate is less than the set threshold, we recover coverage holes by deploying additional sensors in the target regions, i.e., the collection S = { $s_{1}$, $s_{2}$, $s_{3}$, …, $s_{n}$ } needs to be augmented. However, one of the biggest challenges is determining where these additional sensors can be deployed to ensure that the least amount of sensors is used. The challenge can be defined as the Optimum Increase Coverage Problem (OICP), and it is NP-complete. Therefore, in this section, we firstly prove that the OICP is NP-complete and then propose an approximation algorithm to determine the locations of additional sensors. The algorithm is based on Voronoi tessellation and Delaunay triangulation, and we will prove that our algorithm can recover coverage holes using the least amount of sensors by analyzing the theory of Voronoi tessellation and Delaunay triangulation.

#### 5.2.1. The Optimum Increase Coverage Problem (OICP) is NP-Complete

As proved by Zhao et al. [43], the Optimum Partition Coverage Problem (OPCP) is NP-complete. The OPCP aims to find the optimal deployment strategy to deploy sensors in the target regions with the least amount of sensors to achieve full coverage. Similarly, the OICP aims to find the optimal deployment strategy to deploy additional sensors in the target regions with the least amount of sensors to recover existing coverage holes. Thus, the OPCP and OICP are closely connected by the requirement of deploying sensors. If the OICP is not NP-complete, and it can be solved by a specific algorithm, we can deploy sensors on the basis of the algorithm to solve the OPCP. Firstly, a small number of sensors are randomly deployed in the target regions, and we can then recover coverage holes by adding additional sensors. The locations of additional sensors are determined by the algorithm of solving the OICP. Thus, the OPCP is not NP-complete, and the conclusion is completely contradictory with the conclusion in [43]. Therefore, the OICP is NP-complete.

#### 5.2.2. Voronoi Tessellation

**Figure 7:**
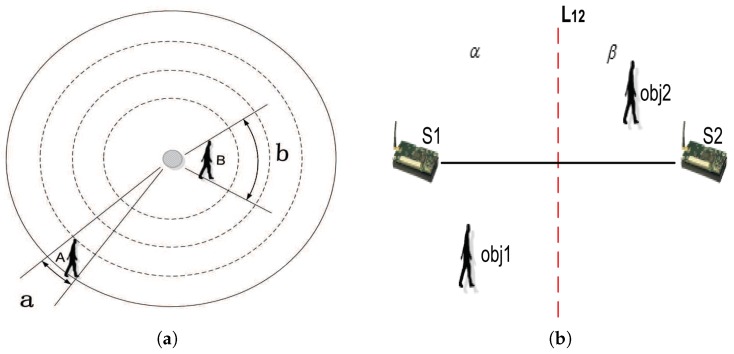
Voronoi tessellation. (**a**) The influence of distance. A and B are two device-free objects with an approximately equal size, and the gray dot denotes a sensor node. (**b**) The theory of Voronoi tessellation. $S_{1}$ and $S_{2}$ are two sensor nodes that can communicate with each other, $L_{12}$ is the perpendicular bisector of $S_{1}S_{2}$ and divides the plane into two regions. For any object in α, the Euclidean distance from it to $S_{1}$ is smaller than it is to $S_{2}$ in terms of the mid-perpendicular theorem.

**Figure 8:**
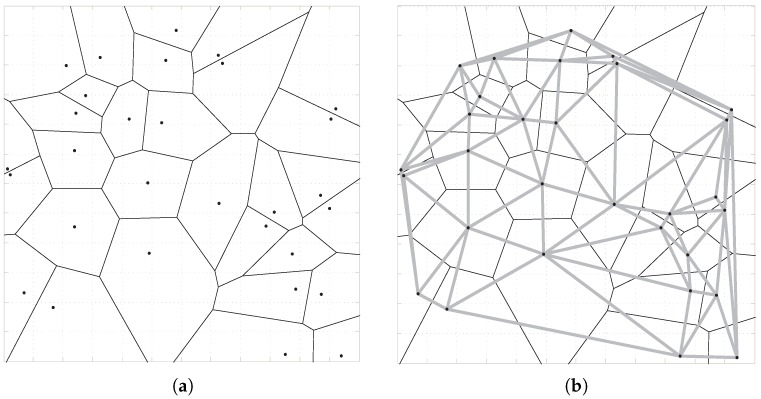
Voronoi tessellation (**a**) and Delaunay triangulation (**b**).

In order to add additional sensors as little as possible, we need to find the sparsest part of the target regions in which additional sensors will be deployed. Note that RSSI values of a sensor node are affected more easily by the nearest object than those far away from it. As shown in Figure 7a, A and B are two device-free objects with approximately equal size, and the gray dot denotes a sensor node. *a* and *b* are the angles of regions disturbed by A and B, respectively. Obviously, the distance from A to the sensor node is greater than it is from B to the sensor node, and B affects more communication links than A. Thus, a two-dimensional plane in which a set of sensors are deployed is considered, and the plane is divided into a number of sub-regions. Each sub-region is a point set in which the Euclidean distance from any point to the sensor node is smaller than it is to all other sensor nodes. The sparsest part of the target regions will be obtained. Thus, Voronoi tessellation is introduced to partition the target regions. An example of Voronoi tessellation is shown in Figure 8a. Next, we describe the process of Voronoi tessellation in detail.

As shown in Figure 7b, $S_{1}$ and $S_{2}$ denote two sensor nodes that can communicate with each other. $L_{12}$ is the perpendicular bisector of $S_{1}S_{2}$ and divides the plane into two regions. Let α denote left regions and β denote right regions. For any object in α (such as $Obj_{1}$ in Figure 7b), the Euclidean distance from it to $S_{1}$ is lower than it is to $S_{2}$ in terms of the mid-perpendicular theorem. Moreover, α contains all points that are nearer to $S_{1}$, namely *Z*($S_{1}$). Similarly, let *Z*($S_{2}$) denote the point set in which each point is nearer to $S_{2}$ than it is to $S_{1}$. In order to show that *Z*($S_{1}$) and *Z*($S_{2}$) is divided by the perpendicular bisector of $S_{1}S_{2}$, let *R*($S_{1}$,$S_{2}$) and *R* ($S_{2}$,$S_{1}$) denote *Z*($S_{1}$) and *Z*($S_{2}$), respectively. Let $Z\left(S_{i}\right)=\underset{i<>j}{⋂}R\left(S_{i},S_{j}\right)$ denote the Voronoi tessellation of $S_{i}$. As a result, $VOR=\sum_{i=1}^{n}Z\left(S_{i}\right)$ denotes the Voronoi tessellation of the target regions, where n is the number of the sensor nodes.

Note that the time complexity of Voronoi tessellation is $O\left(n^{3}\right)$ if using the mid-perpendicular method directly, where n denotes the number of sensor nodes deployed in the target regions. One optimization can be made according to [44]. This paper proposes a geometric transformation that combines the sweep line technique, and the balanced binary tree as the basic data structure to calculate Voronoi tessellation with time complexity is $O\left(nlogn\right)$ in the worst case.

#### 5.2.3. Delaunay Triangulation

The locations of additional sensors are still not determined after Voronoi tessellation, so Delaunay triangulation is introduced to determine the locations.

**Definition 1.** ***(Adjacent Voronoi cells)*** In a region $R^{2}$, two Voronoi cells Z($S_{1}$) and Z($S_{2}$) are adjacent if they have a common border.

According to Definition 1, for all sensor nodes, S = { $s_{1}$, $s_{2}$, $s_{3}$, …, $s_{n}$ } in a region if two Voronoi cells *Z*($S_{1}$) and *Z*($S_{2}$) formed by $S_{1}$ and $S_{2}$, respectively, are adjacent, connecting these two sensor nodes through a straight line. Thus, triangulation of the regions (i.e., Delaunay triangulation) is obtained when traversing all nodes, as shown in Figure 8b.

Note that the Delaunay triangulation should obey the following three principles.

**Theorem** **1.** All triangles are disjointed. This means that these triangles should not have other intersections except endpoints.

**Theorem** **2.** All triangles cannot contain other triangles.

**Theorem** **3.** All triangles must be located inside the regions.

With the three principles of Delaunay triangulation, we begin to consider how to meet the requirement of the minimum number of additional sensor nodes. As shown in Section 6.1.2, coverage holes always appear in the middle of the triangle. We can deploy additional sensors in the centre of the triangle to recover coverage holes as much as possible. More accurately, the new sensor should be deployed in the circum-centre of the triangle with the largest area when the coverage rate of the target regions is less than 90%. When the coverage rate is larger than 90% and less than 100%, the new sensor should be deployed in the barycentre of the triangle with the largest area.

Suppose that the sensors are randomly deployed. Some regions might be so sparse that they cannot satisfy the requirement of coverage rate when only a sensor node is added. Therefore, a recursive method is required. The addition of new sensors will cause the changes in the configuration. Thus, we need to recalculate Voronoi tessellation. In order to reduce the computation complexity, a subgraph can be constructed which consists of the new additional sensors and its surrounding sensors, and the Voronoi tessellation and Delaunay triangulation of the subgraph can be calculated. The pseudocode of recovering coverage holes is shown in Algorithm 2.

**Algorithm 2:** Procedure of Recovering Coverage Holes**Input**: The initial set of sensors in the target regions: $S1=\{s_{1},s_{2},s_{3},\ldots ,s_{n}\}$; The required coverage rate of the specific applications: coverage_rate_req;
**Output**:
 The new set of sensors in the target regions: $S1^{\prime }=\left\{s_{1},s_{2},s_{3},\ldots ,s_{n},\ldots \ldots \right\}$;
 **1**Calculate the coverage rate coverage_rate with Algorithm 1; **2****while** *coverage_rate is not equal to coverage_rate_req* **do** **3**  Voronoi tessellation; **4**  Delaunay triangulation; **5**  Calculate area of all triangles of Delaunay triangulation and find the triangle with the largest area; **6**  **if** *coverage_rate is less than 90%* **then** **7**    Calculate the coordinates of the circum-centre of the triangle: $s_{i}\left(x_{s_{i}},y_{s_{i}}\right)$; **8**    **else** **9**      Calculate the barycentric coordinates of the triangle: $s_{i}\left(x_{s_{i}}^{\prime },y_{s_{i}}^{\prime }\right)$**10**     **end****11**   **end****12**   Add $s_{i}$ to the set of $S1^{\prime }$**13**   Calculate the coverage rate coverage_rate with Algorithm 1;**14** **end**

Now we give the calculations of area and barycentre of a triangle on the premise of knowing three vertexes’ coordinates.

The set of triangles of Delaunay triangulation in $R^{2}$ is denoted as $T=\left\{t_{1},t_{2},\ldots ,t_{i}\right\}$. For any triangle $t_{i}$, suppose its vertex coordinates are denoted as $s_{i}\left(x_{i},y_{i}\right)$, $s_{j}\left(x_{j},y_{j}\right)$, and $s_{k}\left(x_{k},y_{k}\right)$. Then, according to Equation (8), the area of every triangle of Delaunay triangulation can be calculated. The triangle with the largest area can be obtained by a sort algorithm, and its barycentric coordinates $ct_{i}\left(x_{ct_{i}},y_{ct_{i}}\right)$ can be calculated by Equation (9).

(8) $$Area_{t_{i}}=\frac{1}{2}\left|\begin{array}{ccc} x_{i} & y_{i} & 1\\ x_{j} & y_{j} & 1\\ x_{k} & y_{k} & 1 \end{array}\right|$$

(9) $$\left\{\begin{array}{c} x_{ct_{i}}=\frac{x_{i}+x_{j}+x_{k}}{3}\\ y_{ct_{i}}=\frac{y_{i}+y_{j}+y_{k}}{3} \end{array}\right..$$

#### 5.2.4. Algorithm Description and Analysis

For the target regions $R^{2}$, denoted as S = {$s_{1}$, $s_{2}$, $s_{3}$, …, $s_{n}$ }, where *n* is the number of sensor nodes. The approximation algorithm can be described as follows:

1. Firstly, we divide the regions into several small enough cells and verify whether these small cells are covered one by one. Then the total area of covered regions can be calculated by adding up all covered small cells in the target regions. The time complexity of this step is $O\left(n\right)$, where *n* is the number of small cells, which is determined by the size of small cells.
2. We then calculate the coverage rate of the target regions by calculating the ratio of the total area of covered regions with the area of the target regions. If the coverage rate achieves the requirement of the corresponding systems, then exit; if not, then continue.
3. Partitioning the regions by Voronoi tessellation. According to the optimization proposed in [44], the worst case time complexity is $O\left(nlogn\right)$, where *n* is the number of sensors deployed in the target regions.
4. According to the dual graph of Voronoi tessellation, we can obtain Delaunay triangle $T=\left\{t_{1},t_{2},\ldots ,t_{i}\right\}$. Because the common border of adjacent Voronoi cells can be obtained in the process of Voronoi tessellation, the time complexity of the Delaunay triangle is $O\left(n\right)$, where *n* is the number of Delaunay triangles.
5. We will next calculate the total area of all triangles of Delaunay triangulation and find the triangle with the largest area. The time complexity is determined by the sort algorithm, and it is $O\left(nlogn\right)$, where *n* is the number of Delaunay triangles.
6. Finally, the new sensors will be deployed. Go back to Step 2.

## 6. Experiment and Evaluation

In this section, on the one hand, the experimental setup is given to analyze the coverage model in the case of a single link and multiple links, respectively. Table 2 shows the main experimental parameters of the two cases. On the other hand, we assess the performance of the algorithm by conducting simulation experiments. The simulation experiments are performed using MATLAB R2014a, in Windows 10. The size of the target region is 300×300 m. The transmission range and the sensing range of the sensor nodes are 100 m and 50 m, respectively. In addition, we manually deploy sensors on the borders of the target region in order to facilitate comparison.

### 6.1. Experimental Setup

#### 6.1.1. Single-Link Setup

**Figure 9:**
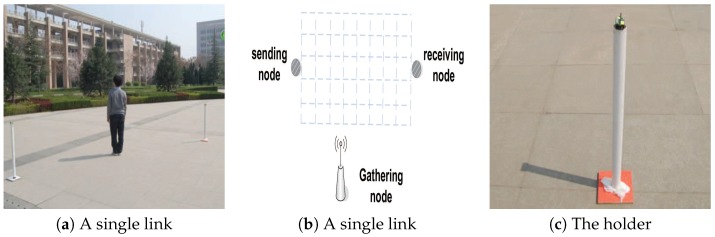
Experimental deployment. (**a**) The experimental deployment when there is only one link. (**b**) The partition of regions when there is a single link. (**c**) The holder for reducing the influence of the ground.

**Figure 10:**
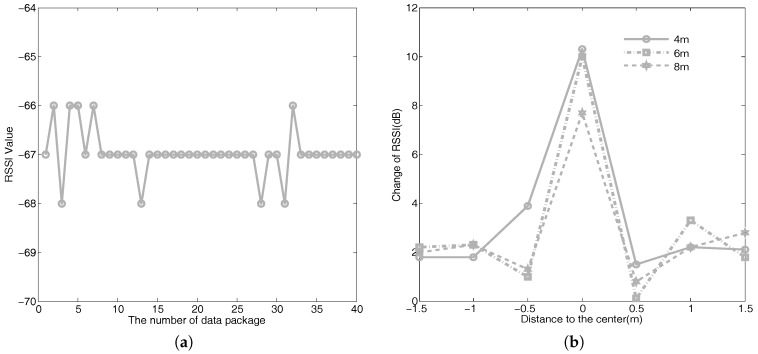
(**a**) RSSI values of one link when there are no objects between two sensors. (**b**) The influence of different distance intervals .

Firstly, two sensors with 4 m distance intervals are deployed in an empty region, as shown in Figure 9a. In order to reduce the influence of the ground, sensors are put in a holder and the height of the holder is 1.5 m, as shown in Figure 9c. Then, as shown in Figure 9b, regions between two sensors are equally divided into 40 small cells with a size of 0.5×0.5 m. We firstly collect RSSI values when there are no obstacles between two sensors. Figure 10a shows the result. The RSSI values are approximately −67 dB, and there are approximately 1 dB fluctuations when there are no obstacles between two sensors. Therefore, we believe that the object cannot be tracked and monitored when the changes of the RSSI values are less than 1 dB. Then we collect RSSI values when a person stands in different cells. The height of the person is 1.75 m, and s/he covers about 0.7×0.3 m. The result is shown in Figure 5a, and the different gray scales stand for different changes in RSSI values. Next, we verify the influence of different distance intervals. Figure 10b shows the changes of RSSI values at distance intervals of 4, 6, and 8 m, respectively. The results show that the distance interval does not affect the ellipse coverage model between two sensors when the interval is within a certain range.

#### 6.1.2. Multi-Link Setup

**Figure 11:**
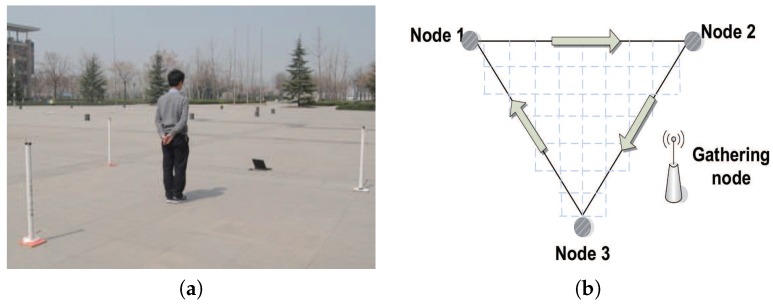
Experimental deployment. (**a**) The experimental deployment when there are three links. (**b**) The partition of regions when there are three links.

As shown in Figure 11a, three sensors are deployed with a distance interval of 4 m in a region and sensors communicate with each other. The regions inside the three sensors are equally divided into 34 small cells with a size of 0.5×0.5 m, as shown in Figure 11b. We firstly collect the RSSI values of three communication links when there are no obstacles, denoted as $R=\left\{r_{raw1},r_{raw2},r_{raw3}\right\}$. Then we collect RSSI values of three communication links when a person with a height of 1.75 m and covering an area of 0.7×0.3 m stands in different cells. Forty RSSI values are recorded for every cell. Let $O_{i}=\left\{r_{1i},r_{2i},r_{3i}\right\}$ denote the RSSI values of cell *i*, where $r_{1i}$, $r_{2i}$, and $r_{3i}$ are the mean values of 40 records of three communication links, respectively. Let $r_{i}$ denote the change in RSSI values of cell *i* and $r_{i}=max\left(\left|r_{1i}-r_{raw1}\right|,\left|r_{2i}-r_{raw2}\right|,\left|r_{3i}-r_{raw3}\right|\right)$. In particular, when $r_{i}>1$, this cell is marked as a covered region. This is because there are approximately 1 dB fluctuations when there are no obstacles. The result is shown in Figure 5b, and the different gray scales stand for different changes in RSSI values.

### 6.2. The Ellipse Coverage Model vs. the Disk Coverage Model

**Figure 12:**
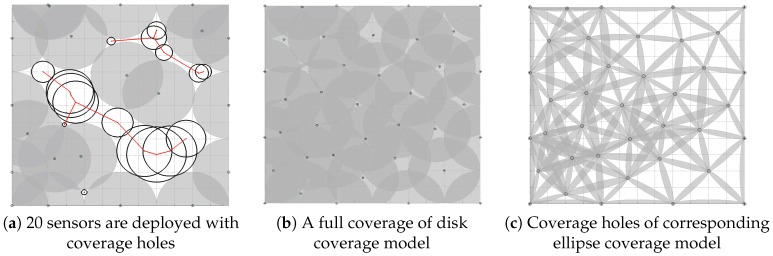
Coverage holes of the ellipse coverage model.

Most existing methods of detecting and recovering coverage holes are based on the disk coverage model. These methods, however, cannot be used to detect and recover coverage holes of WSNs based on the ellipse coverage model. This is because there are still coverage holes for the ellipse coverage model when it is a full coverage for the disk coverage model. Figure 12a shows that 20 sensors are deployed in the target regions and that there are coverage holes. We recover coverage holes according to the method proposed by Wei Li [40]. The method first detects and describes coverage holes according to the theory of trees and graph, and additional sensors are then deployed at the centre of the large empty inscribed circles (IECs). Figure 12b shows that all coverage holes are recovered when 17 additional sensors are deployed. However, there still are coverage holes for the ellipse coverage model, as shown in Figure 12c. Table 3 shows the result when a different initial number of sensors is deployed. Specifically, when 40 sensors are initially deployed, 7 additional sensors are need to recover coverage holes based on the disk coverage model, and the coverage rate can reach 100%. However, the coverage rate based on the ellipse coverage model is only 32.69%, which means that there still are coverage holes for the ellipse coverage model. The result shows that there are always coverage holes for the ellipse coverage model when coverage holes are recovered, according to the methods based on the disk coverage model. Furthermore, the result also proves that the method of recovering coverage holes based on the disk coverage model cannot be used to recover coverage holes based on the ellipse coverage model.

**Figure 13:**
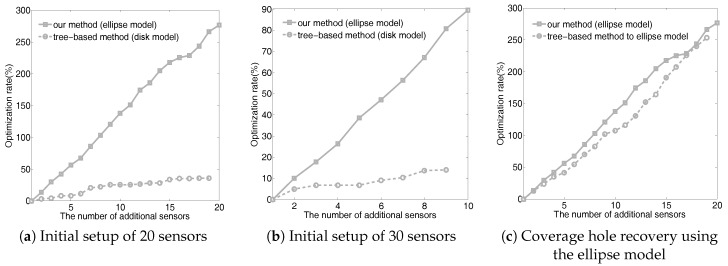
Evaluation setup of 20 (**a**) and 30 (**b**) initial sensors. (**c**) The effectiveness of the recovery methods using the ellipse model. Our approach delivers a higher optimization rate across all scenarios.

Because different coverage models lead to different coverage rates, we used the optimization rate to evaluate the overall performance of our method. The optimization rate was calculated using Definition 2, indicating the contribution of additional sensors. The higher the optimization rate is, the better the recovery method is. Figure 13 shows the relationship between the number of additional sensors and the optimization rate. The baseline is a tree-based method [40] based on the disk coverage model. Our experiments include two initial setups, involving 20 and 30 deployed sensors, respectively. We then added new sensors based on the calculation of our approach and the tree-based method. As can be seen from the diagram, our method has a better optimization rate using the same number of additional sensors and delivers a significantly better performance using fewer sensors. We also use the coverage hole recovery method in [40] to recover coverage holes based on the ellipse coverage model. The relationship between the number of additional sensors and the optimization rate is shown in Figure 13c when 20 sensors are initially deployed. Again, our approach delivers significantly better performance, showing that the previous method is ill-suited for RSSI-based localization.

**Definition** **2.** The ***optimization rate** is calculated as $imp=\frac{Cover_{aft}-Cover_{bef}}{Cover_{bef}}$, where $Cover_{bef}$ and $Cover_{aft}$ are the coverage rate before and after adding new sensors, respectively.*

**Figure 14:**
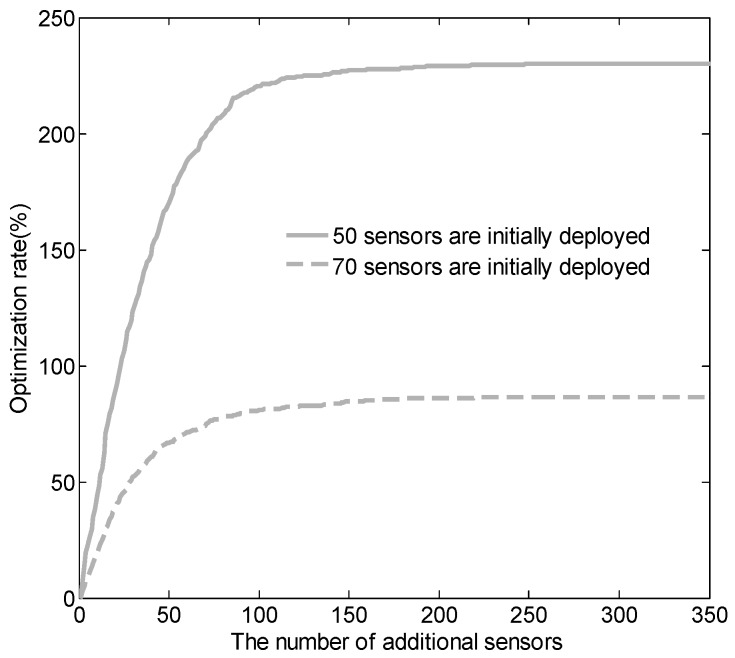
Relationship between the number of additional sensors and optimization rate. We initially deployed 50 and 70 sensors and then added new sensors according to our algorithm until the coverage rate reached 100%.

The optimization rate function, imp, defined in Definition 2, is consistent with the submodilarity theory [45]. Specifically, the improvement of the optimization rate depends on how many sensors have been added—as the number of added sensors increases, the gain in the optimization rate decreases. Figure 14 gives a concrete example of how the optimization rate changes as the number of additional sensors increases. In this example, we first deployed 50 and 70 sensors in the target region, and we then added sensors one by one according to our algorithm until the coverage rate reached 100%. Figure 14 shows the relationship between the number of additional sensors and the optimization rate. As can be seen from the diagram, the optimization rate is a concave function of the number of additional sensors. We also note that the optimization rate is nondecreasing when imp(ϕ)=0. Satisfying both properties mean that our optimization rate function complies with the submodilarity theory.

### 6.3. Results of Coverage Hole Recovery

In this section, the process of the approximation algorithm is illustrated, and the performance of the algorithm is evaluated.

**Figure 15:**
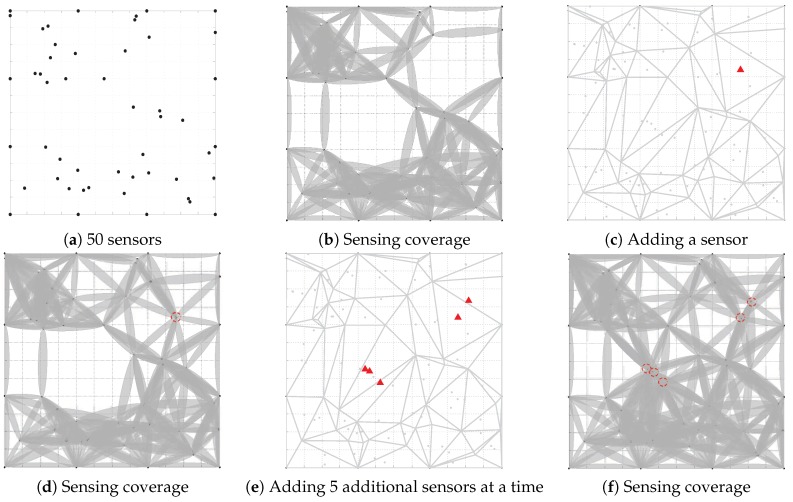
An example of algorithm execution. (**a**) 50 sensors are randomly deployed in the target region. (**b**) The sensing coverage of the network. (**c**) The position of the new sensor. (**d**) The sensing coverage after adding a sensor. (**e**) The positions of the five additional sensors. (**f**) The sensing coverage after adding five sensors.

Figure 15 shows the operational process of our algorithm. As shown in Figure 15a, 50 sensor nodes are randomly deployed in the target regions. The sensing coverage of these sensors is shown in Figure 15b. The coverage rate can be calculated as 30.29%. Figure 15c shows the position of the additional sensor (i.e., the red triangle), and Figure 15d shows the sensing coverage of the regions. Of course, multiple sensor nodes can be added at the same time according to our algorithm. Figure 15e,f show the position of five additional sensor nodes and shows the coverage when five sensor nodes are added. Thus, multiple sensor nodes can be added at a time when the coverage rate is relatively low.

**Figure 16:**
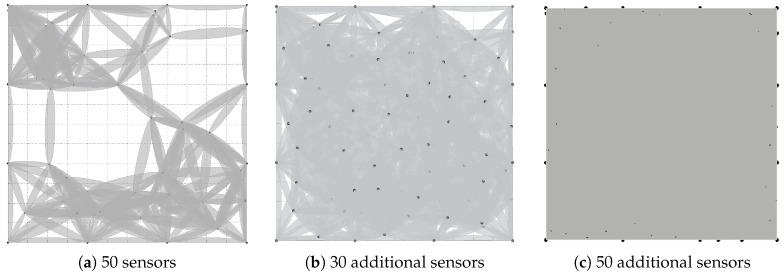
Coverage holes and recovering coverage holes. (**a**) 50 sensors are deployed in the target regions, and there are coverage holes. (**b**) 30 additional sensors to recover coverage holes. (**c**) Recovering coverage holes by adding 20 more sensors.

Figure 16 shows the process of recovering coverage holes. Figure 16a shows the coverage holes when 50 sensors are deployed. It is obvious that there are coverage holes. We recover coverage holes with additional sensors, and the locations of these new sensors are determined by our algorithm. Figure 16b shows the sensing coverage with 30 additional sensors. Figure 16c shows the sensing coverage when adding 20 more sensors.

As far as we know, this is the first paper to detect and recover coverage holes of WSNs based on the ellipse coverage model. Most existing methods of detecting and recovering coverage holes are based on the disk coverage model. As shown in Section 6.2, these methods based on the disk coverage model cannot be used to detect and recover coverage holes of WSNs based on the ellipse coverage model. In our previous work [46], we propose an algorithm to detect and recover coverage holes of WSNs based on the ellipse coverage model. The algorithm is based on Voronoi tessellation and Delaunay triangulation, and the additional sensor is deployed in the barycentre of the Delaunay triangle with the largest area. However, an improvement to the previous algorithm has been made here.

**Figure 17:**
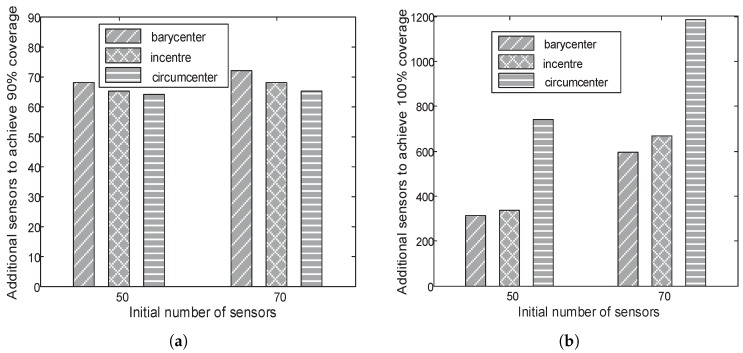
A different number of new sensors are needed when deploying additional sensors in different locations (i.e., the barycentre, the in-centre, and the circum-centre) when the coverage rate is less than 90% (**a**) or the coverage rate is between 90 and 100% (**b**).

**Figure 18:**
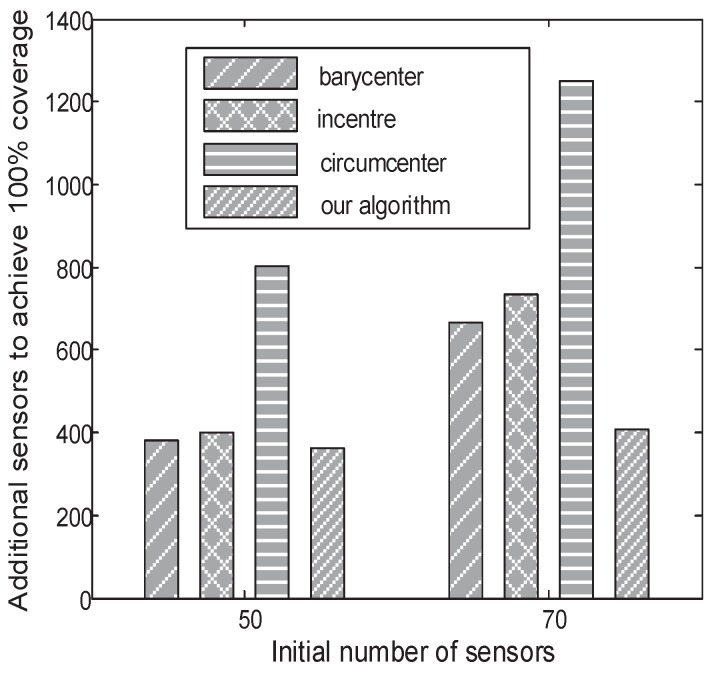
The number of new sensors needed for 100% coverage. When 50 and 70 sensors are initially deployed in the target regions, our method needs 362 and 409 additional sensors, respectively, to achieve 100% coverage, and the method in [46] needs 382 and 664 additional sensors, respectively, to achieve 100% coverage.

Figure 17a shows the number of new sensors to achieve 90% coverage when additional sensors are deployed in different locations (i.e., the barycentre, the in-centre, and the circum-centre) of the Delaunay triangle with the largest area. As shown in Figure 17a, when an additional sensor is deployed in the circum-centre of the Delaunay triangle with the largest area, a minimum number of new sensors are needed. Figure 17b shows the number of new sensors to achieve 100% coverage when the current coverage rate is larger than 90%. Obviously, when the additional sensors are deployed in the barycentre of the Delaunay triangle with the largest area, a minimum number of new sensors is needed. Thus, we enhance the method of [46] by deploying the additional sensors in the circum-centre of the Delaunay triangle when the current coverage rate is less than 90% and by deploying the additional sensors in the barycentre of the Delaunay triangle when the current coverage rate is between 90 and 100%. The reason we use the threshold of 90% is shown in Appendix A. Figure 18 shows that our method needs a minimum number of new sensors to achieve 100% coverage. When 50 and 70 sensors are initially deployed in the target regions, our method needs 362 and 409 new sensors, respectively, to achieve 100% coverage, and the method in [46] needs 382 and 664 new sensors, respectively, to achieve 100% coverage. Obviously, our method can recover coverage holes with the minimum number of new sensors.

## 7. Conclusions

Sensing coverage is one of the most fundamental test indices of the quality of service provided by wireless sensor networks (WSNs). In this paper, we mainly explore the coverage model of RSSI-based localization techniques and enhance RSSI-based localization through coverage hole detection and recovery. Compared to existing studies, it is easy to show that the coverage model of RSSI-based localization techniques is an ellipse determined by two communicating sensor nodes, not a disk centred at a sensor node. We firstly show coverage holes intuitively by performing experiments under realistic conditions and analyze the cause of coverage holes from the perspective of electromagnetic wave propagation theory. Then, an algorithm to detect and recover coverage holes based on Voronoi tessellation and Delaunay triangulation are proposed. Simulation results show that our algorithm can recover coverage holes to reach any coverage rate including 100%.

Our current implementation only considers an ideal deployment scenario with regular shapes and no obstacles. In the experiments, we manually deploy sensors on the borders in advance. We also only consider the situation of scenarios of a 2-dimensional plane. The deployment environment could be more complex in practice. For example, in the application of panda monitoring, some links between some pairs of deployed sensors may be missed because of the influence of trees. Additionally, the target region is not always regular. In future work, we will further improve the algorithm by considering the shape, obstacles, and the borders of the deployment area.

## Figures and Tables

**Table 1:** Notations used in this paper.

$R^{2}$	The target regions
$d_{max}$	The transmission range of sensors
$s_{i}$($x_{i}$, $y_{i}$)	The coordinate of sensor $s_{i}$
*S* = { $s_{1}$, $s_{2}$, $s_{3}$, …, $s_{n}$ }	The set of sensors in the target regions
$d_{ij}$	The Euclidean distance of sensor $s_{i}$ and sensor $s_{j}$
$f_{ij}$	The ellipse coverage model determined by sensor $s_{i}$ and sensor $s_{j}$
$F=\left\{f_{ij}|i,j=1,2,\ldots ,n,i\neq j\right\}$	The set of ellipse coverage model in the target regions
$R^{2}=\left\{a_{1},a_{2},a_{3},\ldots ,a_{m}\right\}$	The set of square elements dividing the target regions
$p_{a_{i}}$	The square element $a_{i}$ is covered
$ℜ=\left\{p_{a_{1}},p_{a_{2}},p_{a_{3}},\ldots ,p_{a_{t}}\right\}$	The set of covered square elements

**Table 2:** Experimental parameters.

Parameter Name	Parameter Value
Node type	Micaz
Distance between nodes	4 m
Height of nodes	1.5 m
Frequency of sending packets	one second every packet
Sampling distance	0.5 m

**Table 3:** The results of different initial number of sensors.

Initial number of sensors	20	30	40	50	60
Additional sensors	17	10	7	5	2
Coverage rate of ellipse model	23.75%	21.05%	32.69%	41.3%	44.83%

**Figure A1:**
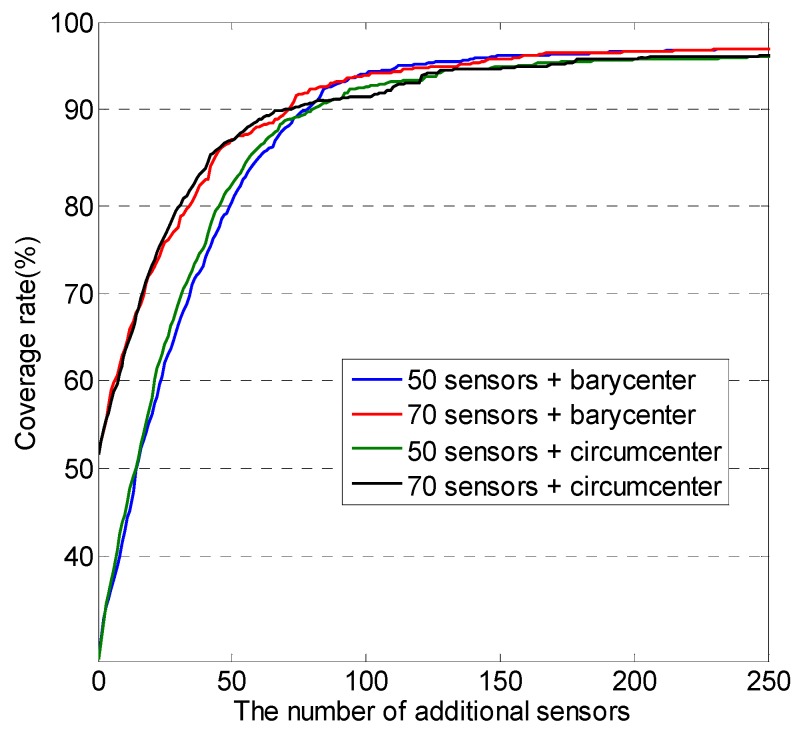
Relationship between the number of new sensors and the coverage rate.

## Appendix A

The appendix explains why a threshold of 90% is chosen to determine where a new sensor should be added to improve the coverage rate. This threshold was chosen based our empirical observations.

Consider two scenarios where 50 and 70 sensors are first randomly deployed in the target regions; next, new sensors are added one by one in the barycentre and the circum-centre of the Delaunay triangle of the largest size. Figure A1 depicts how the number of new sensors affects the coverage rate. As can be seen from the diagram, when the current coverage rate is below 90%, a new sensor in the circum-centre of the Delaunay triangle gives a higher coverage rate. By contrast, adding new sensors in the barycentre of the Delaunay triangle is a better choice when the current coverage rate is above 90%. From this example, we see that the 90% coverage rate is a cut-off point for determining where a new sensor should be added to the target region.
